# Demographic and clinical correlates of depression among older adults with arthritis in Nigeria

**DOI:** 10.4102/sajpsychiatry.v30i0.2264

**Published:** 2024-06-28

**Authors:** Yesiru A. Kareem, Placidus N. Ogualili, Kehinde A. Alatishe, Ismail O. Adesina, Fatima A. Ali, Taiwo A. Alatishe, Richard Uwakwe

**Affiliations:** 1 Directorate of Clinical Services, Neuropsychiatric Hospital, Aro, Abeokuta, Nigeria; 2 Department of Mental Health, Federal Neuropsychiatric Hospital, Maiduguri, Nigeria; 3 Department of Orthopaedics and Trauma, National Orthopaedic Hospital, Igbobi, Lagos, Nigeria; 4 Department of Clinical Services, Federal Neuropsychiatric Hospital, Yaba, Lagos, Nigeria; 5 Department of Psychiatry, LAUTECH Teaching Hospital, Ogbomoso, Nigeria; 6 Department of Mental Health, Faculty of Medicine, Nnamdi Azikiwe University, Nnewi, Nigeria

**Keywords:** demographic, clinical, correlates, depression, older adults

## Abstract

**Background:**

Older adults have a high prevalence of chronic conditions like arthritis with morbidities, especially depression ranging up to 40% – 70%. Therefore, it is important to explore depression in older adults with arthritis.

**Aim:**

This study aimed to determine if any demographic and clinical factors are associated with depression in older adults aged ≥ 60 years with arthritis attending a rheumatology clinic.

**Setting:**

This is a cross-sectional study conducted over 6 months among 127 older adults on follow-up care in a university teaching hospital in the North-Eastern region of Nigeria.

**Methods:**

A clinical proforma with information about the type of arthritis, duration of illness, hospitalisation, use of medications, co-morbidity was utilised for the data collection. The Geriatric Depression Scale (GDS-30), sociodemographic questionnaire and clinical proforma were administered. Data were analysed using Statistical Product and Service Solutions (SPSS) version 26.0 with the level of significance set as 0.05.

**Results:**

The mean age (± standard deviation [s.d.]) was 66.6 (± 5.5) years, with males constituting 57.5%. The prevalence of depression was 57.8%. Osteoarthritis 30.2%, while 69.8% had rheumatoid arthritis. Sociodemographic factors associated with depression include age (*p* = 0.049), marital status (*p* = 0.001), and level of education (*p* = 0.001). Duration of illness (*p* = 0.02), hospitalisation (*p* = 0.03), and number of medications (*p* = 0.01) were clinical factors associated with depression score.

**Conclusion:**

The prevalence of depression in older people with arthritis is high and was associated with females, the widowed, no formal education; and those with long duration of illness, those using multiple medications, and those with repeated hospitalisation.

**Contribution:**

This finding can enhance the suspicion index for depression to establish standard operating procedures, which will help to improve therapeutic practice for caring for the older adult age group.

## Introduction

Older adults are a population of people above 60 years of age. Their population is on the increase because of the advances in medical treatment, with consequent reduction in death on account of communicable and non-communicable diseases.^1,2^ In fact, this decade, namely 2021–2030 has been declared as the decade of healthy ageing by the World Health Organization (WHO).^3^ The effects of ageing on the body tissues, such as degenerative changes in virtually all body tissues pose certain specific medical challenges to the older adults, and this often comes with special medical needs.^4^

Arthritis is a disabling condition, and often refers to a group of disorders associated with damage to the joints and surrounding tissues. It is a leading cause of disability globally, especially among the older adult population.^5^ The Global Burden of Disease (GBD) study in 2019 revealed that depression and musculoskeletal diseases account for a significant percentage of the burden of diseases, constituting among the leading 10 disorders accounting for the Disability Adjusted Life Years (DALYs) worldwide.^6^ Gureje et al. in a study conducted among older adults in the Yoruba speaking area of Nigeria found arthritis to be the most prevalent chronic medical condition, with a prevalence of about 70%.^7^ Many types of arthritis have been described in medical literature, but the most common forms are osteoarthritis (OA) because of degenerative changes, and rheumatoid arthritis (RA) on account of inflammation that is not a reaction to joint infection or injury.^5^ Also, of importance among the older adults is gout, a form of arthritis associated with deposit of uric acid in the joints.^8^ The disabilities associated with arthritis are usually because of chronic, often unbearable pain and stiffness of the joints, most times leading to limitation of movement and use of the affected joints.^9^

Older adults that have arthritis, just like other chronic medical conditions, are at risk of developing neuropsychiatric morbidities. Depression has been described as the most common neuropsychiatric morbidity associated with this group of people.^10^ Depression as a disorder has been described as being associated with greater disability than chronic medical conditions like diabetes, asthma and arthritis in older adults.^11^ Its co-occurrence in older adults thus implies greater disability compared to those that have arthritis without depression.^7^ Individuals with arthritis have been reported to have higher risk of depression compared with those who do not have arthritis. A World Health Survey conducted by the WHO in 60 different countries reported that the prevalence of depression among participants that have arthritis was about three times the prevalence in those who did not have arthritis.^2^

The relationship between depression and arthritis in older adults is multidirectional. First of all, the excruciating pain, stiffness and limitation of functioning associated with arthritis can lead to frustration, negative thoughts and reduced quality of life, which can result in depressed feelings and depressive disorder with time. Medications used to treat arthritis, like steroids have also been identified as risk factors for developing depression.^10^ In addition, inflammatory mediators like interleukin-6 and tumour necrosis factor alpha have been found to play a role in the development of both arthritis and depression.^12^ Furthermore, Wang et al. in a symptomatic knee osteoarthritis prediction model found that individuals with depression had two or three times risk of developing knee osteoarthritis compared to those who did not have depression.^13^ Arthritis, being a chronic medical condition has also been found to influence the course and outcome of depression. In the same vein, presence of depression has been reported to increase pain sensitivity, thus increasing the perceived intensity and duration of pain in those with arthritis.^14^

Sociodemographic characteristics such as patient gender, level of education, employment status have been recognised as likely determinants of depression in individuals with rheumatoid arthritis.^15^ Also, Chimbo et al., 2022 reported a link between advancing age and development of depression in individuals with RA. Also, duration of arthritis has been reported as being associated with depression.^16^ Furthermore, the pathophysiology of depression in RA also involves pro-inflammatory cytokines such as interleukins −1, −6, and −18.^17^ Even though the pathophysiology of the correlates of depression in OA is unclear, the duration, number of sites and (slow) gait have been implicated.^18^

Occurrence of depression in older adults with arthritis has been found to be associated with poorer quality of life, poorer functioning and response to treatment, higher economic burden and increase in mortality.^19^ Despite this, the mental healthcare needs of individuals in this age group have not been given sufficient attention. Depression among them is often under-diagnosed and under-treated. This is made worse by paucity of healthcare resources in low- and middle-income countries (LMIC) like Nigeria. In the North-eastern part of Nigeria, even more competing interests like victims of terrorism and internally displaced persons receive more attention in allocating the available health care resources.

This study in North-eastern Nigeria draws attention to the prevalence of depression, and the sociodemographic and clinical factors that are associated with depression in older adults with arthritis in this region. It has a possible overreaching impact that may be achieved to increase awareness and management of depression among older adults with arthritis.

This study aimed to estimate the prevalence and severity of depression, and to determine the demographic and clinical correlates associated with depression in older adults with arthritis attending the rheumatology clinic.

## Research methods and design

This research is a cross-sectional study conducted in the speciality clinics in the Maiduguri Metropolis, Borno State. Consecutive older adults ≥ 60 years who presented at the rheumatology clinic in the study location University of Maiduguri Teaching Hospital (UMTH), Maiduguri, Nigeria, were recruited over a 6-month period. The G*power version 3.1 software 19 was employed for the sample size estimation.^20^ Using a prevalence of 35% adopted from a similar study for depression among older adults in Damaturu, a bordering city to the study location using Geriatric Depression Scale (GDS)-30,^21^ a power of 85% and a level of significance of 0.05 were chosen for the sample size calculation. An assumption of a 90% response rate was made, while an estimated non-response rate of 10% was corrected. Thus, a sample size of 127 was used for the study.

Rheumatologists made the diagnosis of arthritis, and these diagnoses were retrieved from the medical records.^22^ The socio-clinical characteristics of the participants were obtained using a sociodemographic questionnaire and a clinical proforma which are investigator-administered tools developed by the authors. Meanwhile, the GDS-30 was used to assess depression. It is a 30-item self-report scale designed specifically for the older adult group. It has been validated and used to screen depression among older adults both internationally^23^ and locally.^24^ A previous study by Sokoya on geriatric depression among Nigerian older adults of ages 60 and above attending primary care found a sensitivity of 84% and 95% specificity.^13^ Scoring of GDS-30 is dependent on the answer given with the highest possible score of 30 points. Scores on the scale are graded as: normal (0–9), mild (10–19), and severe depression (20–30).^25^ The above instruments were translated into the Hausa language using the WHO’s iterative back-translation method to prevent alteration for those who do not understand English. This translation was done by indigenous speakers who understood the Hausa language well and were proficient in English.

Data collected were subjected to integrity and consistency tests before coding. Data collected were then double entered into the Statistical Product and Service Solutions (SPSS) version 26 using a well-defined codebook.^26^ Two-tailed tests were conducted in the analyses, and a *p*-value < 0.05 was considered significant. The univariate analysis measured the independent variables (age, gender, marital status, education, illness duration, medications, and hospitalisation) and the dependent variable (depression classified into no, mild and severe depression). Data were first tested for normality using the Kolmogorov-Smirnov test and then for equality of variance using the Levene test of variance. Where the data did not follow a normal distribution, a non-parametric test of Mann-Whitney U was utilised for two group variables (gender, co-morbidity) and the Kruskal Wallis H test for ≥ 3 groups (marital status, education, duration, medications, and hospitalisation) to compare the median scores across these independent variables. The higher the GDS-30 depression score, the more the severity of the depression.

### Ethical considerations

This study was conducted after obtaining ethics approval from the Research and Ethics Committee (REC) of the study site UMTH with ethical clearance number UMTH/REC/21/779. The purpose, procedure, benefits, and other information on the study were explained to the participants to obtain their written informed consent. The information sheet explained the research procedure, while international ethical norms and standards were strictly adhered to. This procedure was used to ensure adherence to ethical guidelines on informed consent, and the protocol is consistent with the principles of the 1964 Declaration of Helsinki and its later amendments in 2013.

## Results

A total of 127 older adults participated in the survey. The age range was 60–82 years, with a median age (interquartile range [IQR]) of 66.0 (± 8.0) years (see Table 1). The mean age (± standard deviation [s.d.]) was 66.6 (± 5.5) years. About 85 (66.9%) participants were aged between 60 and 69 years, with male predominance constituting 73 (57.5%).

**TABLE 1:** Sociodemographic profile of participants.

Variables	*n*	%
**Gender**		
Male	73	57.5
Female	54	42.5
**Age group (years)**		
60–69	85	66.9
70–79	36	28.4
≥ 80	6	4.7
**Marital status**		
Married	82	64.6
Divorced	17	13.4
Widowed	28	22.0
**Current living arrangement**		
Living alone	13	10.2
With spouse	35	27.6
With children	49	38.6
With relatives	30	23.6
**Family type**		
Monogamous	45	35.4
Polygamous	82	64.6
**Highest level of education**		
No formal education	47	37.0
Quranic only	27	21.3
Primary	22	17.3
Secondary	16	12.6
Tertiary	15	11.8

Among the 127 participants, osteoarthritis was present in 38 (29.9%), while 89 (70.1%) had rheumatoid arthritis (see Table 2). Of the total participants, 72 (56.7%) have been suffering from arthritis for 10 years or less, with 77 (60.8%) previously admitted for medical condition, while 65 (51.2%) were using between 3 and 4 medications for the arthritis.

**TABLE 2:** Clinical characteristics of participants.

Variables	*n*	%
**Type of arthritis**		
Osteoarthritis	38	29.9
Rheumatoid arthritis	89	70.1
**Duration of condition (years)**		
1–5	34	26.8
6–10	38	29.9
11–15	22	17.3
16–20	25	19.7
≥ 21	8	6.3
**Number of medications used**		
1–2	2	1.6
3–4	65	51.2
≥ 5	60	47.2
**Number of previous hospitalisation**		
None	50	39.4
1–2	54	42.5
3–4	22	17.3
≥ 5	1	0.8
**Family history of medical illness**		
No	40	31.5
Yes	72	56.7
Do not know	15	11.8

**FIGURE 1:**
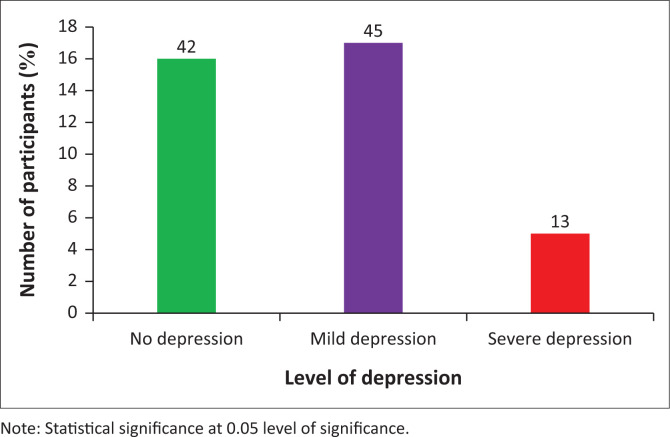
Prevalence of depression among participants with osteoarthritis.

**FIGURE 2:**
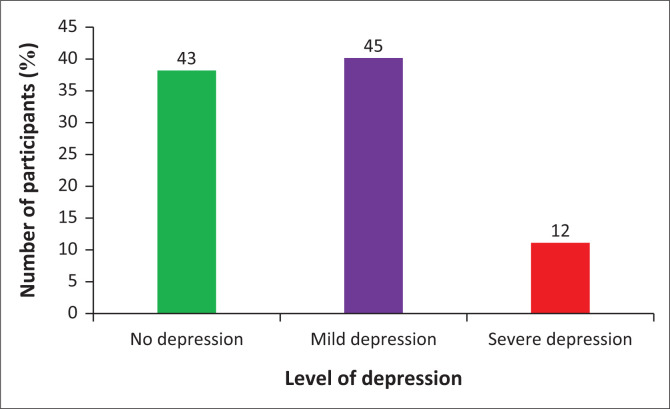
Prevalence of depression among participants with rheumatoid arthritis.

The rates of depression in the different populations with osteoarthritis and those with rheumatoid arthritis are as shown in Figure 1 and Figure 2.

Table 3 shows the sociodemographic correlates of depression in the study. Depression was more common among the age group 70–79 years with median GDS score (and H value) of 13.0 (6.024), and a barely significant *p*-value = 0.049. The widowed had the median GDS score (and H value) of 13.0 (48.047) and *p*-value = 0.001; and those with no formal education had the median GDS score (and H value) of 12.5 (43.630) and *p*-value = 0.01.

**TABLE 3:** Sociodemographic correlates of depression among participants with arthritis.

Variables	GDS score		H	U	*p*
	Median	IQR			
**Gender**	-	-	-	11151.0	0.025*
Male	9.0	10.0	-	-	-
Female	12.0	5.0	-	-	-
**Age group (years)**	-	-	6.024	-	0.049*
60–69	10.0	9.0	-	-	-
70–79	13.0	6.0	-	-	-
≥ 80	7.0	0.0	-	-	-
**Marital status**	-	-	48.047	-	0.01*
Married	9.0	6.0	-	-	-
Divorced	12.0	11.0	-	-	-
Widowed	13.0	3.0	-	-	-
**Highest level of education**	-	-	43.630	-	0.01*
No education	12.5	10.5	-	-	-
Quranic	8.0	7.0	-	-	-
Primary	11.0	4.0	-	-	-
Secondary	12.0	10.0	-	-	-
Tertiary	8.0	11.0	-	-	-

* , statistical significance at 0.05 level of significance.

GDS, Geriatric Depression Scale; IQR, interquartile range; U, Mann-Whitney U statistic, H, Kruskal-Wallis H statistic.

Table 4 shows the clinical correlates of depression in the study. Geriatric Depression Scale scores were also significantly higher among those with the longest duration of the medical condition for 21 years and above, with a median of 16.0 and *p*-value of 0.020; those taking five or more medications with a median of 13.0 and *p*-value of 0.001; and those with 3–4 admissions with a median of 14.0 and *p*-value of 0.003.

**TABLE 4:** Clinical correlates of depression among participants with arthritis.

Variables	GDS score		H	*p*
	Median	IQR		
**Duration of condition**	-	-	11.662	0.020*
1–5	12.0	7.0		-
6–10	10.0	6.5	-	-
11–15	11.0	8.0	-	-
16–20	9.0	10.0	-	-
≥ 21	16.0	4.0	-	-
**Number of medications for arthritis and medical conditions**	-	-	56.377	0.01*
1–2	6.5	4.5		-
3–4	8.0	6.0	-	-
≥ 5	13.0	5.8	-	-
**Number of previous hospitalisation for arthritis and medical conditions**	-	-	8.912	0.003*
None	9.0	3.0	-	-
1–2	12.0	9.0	-	-
3–4	14.0	8.0	-	-

* , statistical significance at 0.05 level of significance.

GDS, Geriatric Depression Scale; IQR, interquartile range; H, Kruskal-Wallis H statistic.

## Discussion

This study assessed the prevalence of depression and its sociodemographic and clinical correlates among older adults with arthritis attending the rheumatology clinic.

Our study shows that depression rate is high among patients with arthritis with a rate of 57.8% (25.8% for OA, and 32.0% for RA). This may be because of the effects of monoaminergic neurotransmission and the production of neurotrophic factors in arthritis. Also, some of the musculoskeletal clinical features of arthritis, such as fatigue, chronic pain^27^ and functional limitations may be linked with depression too; and may have both confounding and contributory effects on depression in rheumatoid arthritis.^28^ Similarly, the chronic stress associated with managing these conditions can also contribute to depression. The depression rate found in this study is comparable with a similar study by Chimbo et al. who showed a prevalence of depression at 27.6% among the participants with osteoarthritis.^16^ Our study finding here is, however, higher than that of Abdel-Nasser et al. in Egypt who reported 10.0% prevalence.^29^ It is also higher than the first work on geriatric depression in Nigeria by Sokoya et al. which found 7.4% prevalence in primary care attendees. This may be because of the use of different assessment tools than the GDS used in this study.

The age distribution of patients in this study showed that patients were between the age of 60 and 82 years. The median age (IQR) was 66.0 (8.0) years. A large proportion (67%) of patients were 60–69 years old, while about 6 in 10 (57.5%) of them were males. Generally, the males in the North-eastern region present more at the hospital partly because of socio-cultural reasons because they have the opportunity to move around, unlike the females, who need permission to do so, and thus may be relatively isolated.^30^

Depression was more common among the females than the males in the study. This concurs with the WHO report that women are 2–3 times more likely than males to suffer from depression.^26^ Numerous factors can explain why depression is more common among females in this study, including biological, psychological, and social factors. For example, hormonal changes in the post-menopausal period may contribute to depression in women. Furthermore, women may face more stress because of caregiving for their husbands and even some relations, and it can also be from the pressure from other societal and cultural expectations. The participants aged 70–79 had the highest median depression score, hence the most depressed, while those aged 80 and above were the least depressed. While this may be explained by the attainment of Eric-Erickson’s stage of ego integrity versus despair among the old,^32^ other studies showed a higher prevalence of depressive symptoms with increasing age.^33,34^

Although our study found a relationship between age and depression, some other studies did not. These include the study by Majekodunmi et al. who did not find a significant relationship between the age of the respondents and depression^25^ and the study by Baiyewu et al. who noted that age was not significantly associated with an increased prevalence of depression in either Yoruba or African Americans. However, older African Americans do have some higher rates of severe depression.^35^

In this study, the widowed were also more depressed than the others, while the least depressed were the married. This is comparable with the observation of Gureje et al.^36^ in the epidemiology of major depressive disorder in older adult Nigerians in the Ibadan Study of Ageing, in which they reported that being widowed, separated, or divorced was strongly associated with an increased lifetime risk for major depressive disorder.^7^ A possible reason for this can be the social support provided by the spouse. Also, spousal emotional support can help cope with stress and negative emotions. Additionally, having a sense of purpose and responsibility to their partner can increase feelings of fulfilment and happiness. Furthermore, being in a committed relationship can provide a sense of security and stability, which can reduce depression.^33^

The participants with tertiary education were the least depressed in this study. This may be because individuals with higher educational attainment are more enlightened with respect to their health and can better understand the disease state and instructions given concerning healthy living and drug usage.^37^ It could also be because of their relatively better socio-economic status. A longer duration of illness (≥ 21 years), a larger number of hospital admissions (3–4) and a greater number of medication usage (≥ 5) were clinical factors associated with depression. The association of multiple admissions with depression is significant in this study and may be a pointer to the severity of the condition(s) for being hospitalised. A study by Baiyewu et al. on ‘a global perspective of inequalities and inadequacies in the mental health care of older adults’ found that depression is associated with more nursing home care and hospital admissions.^4^

In line with a previous study by Mohammed et al. in Maiduguri, the finding of longer duration of illness being associated with depression in this study conforms with that of the previous finding of depression among the patients of a general hospital.^38^ This may be explained by the fact that dealing with a prolonged illness can have a significant impact on mental health.^39^ The long duration of the illness can cause room for negative thoughts and emotions to surface. Also, the sense of isolation and helplessness that often accompany chronic illness can increase the risk of depression.

### Study limitations

The study was limited because it was hospital-based and employed screening tools. Therefore, diagnostic instruments such as the Mini International Neuropsychiatric Interview (MINI) and Composite International Diagnostic Interview (CIDI) were not used which would have enhanced the credibility of the depression found in the study. Also, the cross-sectional study design was adequate for detecting associations; however, it could not permit the determination of the direction of causality between the outcome and explanatory variables. In addition, a multivariate analysis model was not fitted to control for confounders. However, the study is strengthened by the considerably adequate sample size, while the tools used are internationally recognised and valid. Finally, a study of such magnitude involves participants with heterogeneous people with different backgrounds.

These limitations have implications on the findings of this study. For example, the cross-sectional design is faced with an inability to establish causality while the hospital-based nature sets a caution on generalising the findings for the populace. These call for a need for community-based longitudinal studies to examine the causal links and more predictive value of the factors investigated.

## Conclusion and recommendations

This study found that depression is common among older adults with arthritis, with increased prevalence and severity among those with rheumatoid type, and that sociodemographic factors like female gender, age between 70 and 79 years, having Western education and being widowed were associated with depression. Also, clinical factors like longer duration of illness, larger number of hospital admissions and higher number of medications were associated with depression among older adults with arthritis.

In addition, age, marital status, and education are sociodemographic factors that affect the development of depression among older adults with arthritis, while medications, hospitalisation and duration of the condition are some clinical factors associated with depression among older adults with arthritis.

We thereby recommend a proactive routine use of assessment tools in rheumatology clinics to screen older adults with arthritis to raise the index of suspicion for depression for prompt diagnosis. Also, there should be policy formulation to enhance this practice for early intervention to address the identified factors associated with depression in this study. Furthermore, there should be more extensive and longitudinal research works to support the relationships discovered in this study and possibly establish causality. Finally, there is a need for interdisciplinary team management that might involve a geriatric psychiatrist, a rheumatologist, clinical psychologist, and a social worker to liaise and collaborate effectively to provide diverse perspectives in working on the findings from this study.
